# Influence of Isolation Techniques on the Quality of Plasma Samples: Implications for Cancer Biobanking

**DOI:** 10.3390/ijms262110281

**Published:** 2025-10-22

**Authors:** Francesca Piccotti, Fiorella Treviso, Carlo Morasso, Nadia Pittatore Leone, Antonella Navarra, Sara Albasini, Arianna Bonizzi, Ilaria Tagliolini, Francesca Gorgoglione, Fabio Corsi, Marta Truffi

**Affiliations:** 1 Istituti Clinici Scientifici Maugeri IRCCS, via S. Maugeri 10, 27100 Pavia, Italy; francesca.piccotti@icsmaugeri.it (F.P.); fiorella.treviso@icsmaugeri.it (F.T.); carlo.morasso@icsmaugeri.it (C.M.); nadia.pittatoreleone@icsmaugeri.it (N.P.L.); antonella.navarra@icsmaugeri.it (A.N.); sara.albasini@icsmaugeri.it (S.A.); arianna.bonizzi@unimi.it (A.B.); fabio.corsi@icsmaugeri.it (F.C.); 2 Department of Biomedical and Clinical Sciences, Università Degli Studi di Milano, 20157 Milano, Italy; ilaria.tagliolini@unimi.it (I.T.); francesca.gorgoglione@unimi.it (F.G.)

**Keywords:** biobanking, liquid biopsy, plasma isolation technique, pre-analytical variable

## Abstract

Biobanks are essential for precision oncology, providing high-quality materials for biomedical research. Liquid biopsy has become a key tool for non-invasive detection of tumor-derived biomarkers, including circulating tumor DNA, proteins, and extracellular vesicles. However, the reliability of these assays critically depends on standardized preanalytical procedures. In this study, we evaluated the impact of two plasma isolation methods—direct centrifugation (DC) and density gradient centrifugation (DGC)—on the overall quality of breast cancer samples collected at the Bruno Boerci Biobank (Maugeri, Italy). Plasma obtained with the two methods was analyzed by spectrometry for hemolysis and lipemia, biochemical analysis for protein and lipoprotein composition, flow cytometry for cellular debris and platelet contamination. Preanalytical nonconformities due to hemolysis, icterus, and lipemia were comparable between methods. However, DGC was associated with a higher platelet contamination and reduced albumin and cholesterol levels. Inter-individual variability was preserved, supporting the robustness of patient-specific molecular signatures, despite absolute discrepancies. This study highlights the pivotal role of the isolation techniques in shaping the quality and overall composition of plasma samples. Harmonized, “fit-for-purpose” biobanking protocols are required to ensure reproducibility of downstream analyses, support biomarker discovery, and ultimately advance the identification of novel therapeutic targets in cancer.

## 1. Introduction

Biobanks play a pivotal role in advancing biomedical research by providing high-quality biological samples for various research studies [1,2]. The integrity of the biobanked samples is particularly critical for plasma, which is widely used in molecular assays. In recent years, interest in circulating tumor biomarkers has grown, as they enable early cancer detection, disease monitoring, and therapeutic stratification [3]. Liquid biopsy has emerged as a key tool in precision medicine, offering a non-invasive approach for personalized diagnostics and treatment of cancer [4]. Ensuring plasma quality is therefore essential for the reproducibility and reliability of scientific findings in oncological research [5,6].

In this context, the scientific community and key organizations, such as the International Society for Liquid Biopsy (ISLB) and the International Society for Extracellular Vesicles (ISEV), have issued warnings highlighting the importance of standardizing sample collection, handling, and analysis procedures [7,8]. Such standardization is essential to minimize variability across studies and enhance the robustness of research outcomes aimed at measuring and profiling analytes in biofluids. Preanalytical factors, including the type of collection tube, temperature conditions and time elapsed between blood collection and centrifugation, are largely known as variables influencing plasma quality [9,10,11]. Delays in centrifugation can result in cell lysis, leading to hemoglobin and electrolytes contamination. Similarly, temperature fluctuations can degrade proteins and enzymes, affecting biomarkers’ stability [12,13]. The separation process is also a critical, though often underestimated, step in the preparation of plasma and blood derivatives, as the quality of the separated products can profoundly influence downstream molecular analyses. Recent guidelines [7] increasingly emphasize the need to explicitly report the type of separation method used and to verify key quality parameters, including residual platelet content, lipoprotein levels, and the degree of hemolysis, to ensure reproducibility and reliability in studies involving circulating biomarkers.

This study aims to investigate how different isolation techniques affect the overall quality of plasma samples. The selection of two specific protocols was guided by the standard operating procedures (SOPs) currently in place at the Bruno Boerci Biobank and used to support clinical research protocols running at our institute.

Generally, plasma is predominantly isolated through direct centrifugation (DC), a fast and streamlined method suitable for routine processing [14]. However, in specific protocols where the concurrent isolation of patient’s peripheral blood mononuclear cells (PBMCs) is required, a step of density gradient centrifugation (DGC) is performed on fresh blood samples [15]. This method in principle allows for the simultaneous recovery and cryopreservation of both PBMCs and plasma from the same blood sample, as the plasma, being less dense, forms a distinct upper layer above the PBMCs and the Ficoll phase during centrifugation. DC and DGC isolation techniques differ in several key aspects: while DC involves a straight centrifugation step at high speed, DGC requires layering the blood over a density gradient medium followed by centrifugation at lower speed, with no braking or acceleration, in order to preserve the integrity of the cell layer formed at the plasma–gradient interface.

By comparing DC and DGC methods, we aimed to assess plasma quality and evaluate whether DGC represents an appropriate and reliable alternative, particularly in protocols requiring the simultaneous biobanking of both plasma and PBMCs from a single blood draw. Key preanalytical variables such as hemolysis, lipemia, and icterus were analyzed in both series of plasma, as common indicators of sample integrity. Cell debris and residual platelets were also analyzed, as along with major electrolytes and the profile of protein and lipoprotein components to define the overall composition of the plasma obtained with the two methods.

## 2. Results

### 2.1. Preanalytical Quality Indices

Peripheral blood samples (approximately 6–10 mL each) were collected from 50 breast cancer patients and divided into two aliquots (3–5 mL per aliquot) for subsequent plasma isolation using two different methods in a comparative study. For each blood sample, two plasma aliquots were obtained, one isolated with DC and one with DGC, resulting in a total of 100 samples.

We first observed that the volume of plasma recovered following whole blood centrifugation was lower in the DGC group (44.91% of initial blood ± 7.06) compared to the DC group (54.41% ± 7.95; *p* < 0.0001; Supplementary Figure S1). These data correspond to a 17.4% relative reduction in total plasma yield when using DGC vs. DC method.

**Figure 1:**
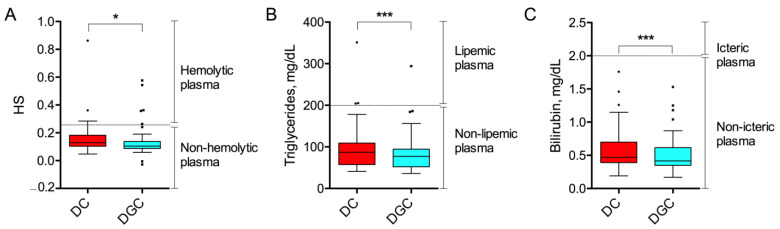
Pre-analytical factors measurement. Plasmatic hemolysis score (HS) (**A**), triglycerides (**B**) and bilirubin (**C**) concentration were measured in DC and DGC groups (n = 50). The dashed lines represent the thresholds for distinguishing hemolyzed (HS > 0.25), lipemic (fasting triglycerides > 200 mg/dL), and icteric (bilirubin > 2 mg/dL) samples. Data are shown as box and whisker plots. Each data point represents an individual plasma sample analyzed. Each box represents the area between the 25th and 75th percentiles [±1.5 IQR]. Lines inside the boxes represent the median values. * *p* < 0.05; *** *p* < 0.0001 by Wilcoxon test.

Hemolysis score (HS) was slightly lower in DGC samples (0.14 ± 0.11, 95% CI [0.10–0.17]) as compared to DC (0.16 ± 0.12, 95% CI [0.12–0.19], *p* = 0.01, Figure 1A). In general, the majority of samples, i.e., 91/100, were classified as non-hemolytic, with an HS lower than 0.25 [16,17]. In contrast, 9/100 samples failed to meet the acceptable range and were categorized as hemolytic. Overall, the frequency of hemolyzed samples was found to be comparable between the two separation methods, with 4 hemolyzed samples (8%) in DC group and 5 (10%) in DGC group (*p* = 0.81, Table 1).

As a measure of lipemia, we considered the plasma concentration of triglycerides, which represents the primary type of fat present in the blood. Mean values were 97.22 mg/dL (±54.25, 95% CI [81.64–112.8]) and 85.12 mg/dL (±46.76, 95% CI [71.69–98.55]) in DC and DGC group, respectively (*p* < 0.0001, Figure 1B). Most of the samples in both groups were found to be within the normal range for fasting triglycerides, which was set at values < 200 mg/dL, hence classified as non-lipemic [18]. Only 3 lipemic samples (6%) were found in the DC group and 1 (2%) in the DGC group (*p* = 0.28, Table 1). Icterus was evaluated by measuring the plasmatic bilirubin concentration. Results indicated a mean value of 0.58 mg/dL (±0.31, 95% CI [0.49–0.67]) for DC and 0.52 mg/dL (±0.27, 95% CI [0.44–0.59]) for DGC samples (*p* < 0.0001, Figure 1C). Icterus was defined in case of bilirubin > 2 mg/dL [19] and was not observed in any of the analyzed samples in both DC and DGC groups.

### 2.2. Cellular Debris, Platelets, and Biochemical Composition

**Figure 2:**
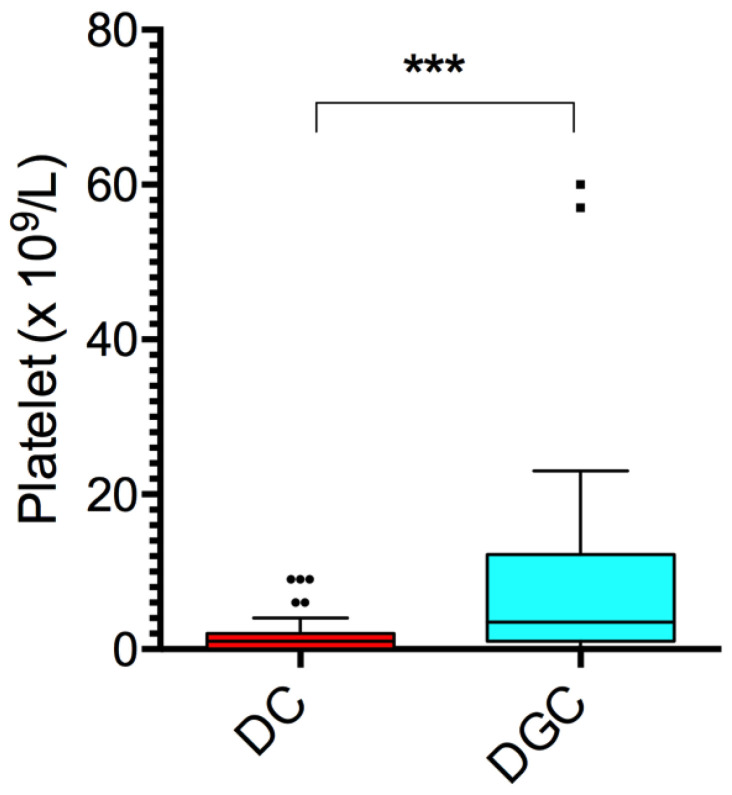
Plasmatic platelets levels measured in DC and DGC samples (n = 50). Data are shown as box and whisker plots as described above. *** *p* < 0.0001 by Wilcoxon test.

The presence of cellular debris was a critical parameter in evaluating the efficacy of the separation methods aimed at isolating cell-free plasma from whole blood. No residual red or white blood cells were detected in any of the samples processed using either the DC or DGC method, indicating effective removal of these cellular components in 100% of cases. In contrast, flow cytometry analysis revealed that platelet contamination was present in a subset of samples. Overall, the DC method yielded a lower median platelet count (1.0 × 10^9^/L, IQR [2.0]) compared to the DGC method (3.5 × 10^9^/L, IQR [11.2]; *p* < 0.0001, Figure 2). In the majority of cases, the levels of residual platelets remained below 10 × 10^9^/L, thus confirming the efficiency of both methods in depleting most of the platelets circulating in the whole blood. However, differences in distribution patterns reflected a significant impact of the isolation method on the level of residual platelet content (see Supplementary Figure S2).

Then, the level of some electrolytes was measured to assess the quality of the samples and to compare the two separation methods. Calcium and potassium levels were not reliable measurements in our context, as the blood collection was performed in K3EDTA tubes as per SOPs. Indeed, as expected, total calcium level was below the detection limit, i.e., <1.0 mg/dL, while potassium was >10.0 mmol/L in both sample series. Magnesium levels were also significantly affected by the presence of the chelating agent in the collection tubes, often resulting in values below the reference range in both DC and DGC groups; hence, calcium, potassium and magnesium were not considered valid parameters for comparison between the two methods.

**Figure 3:**
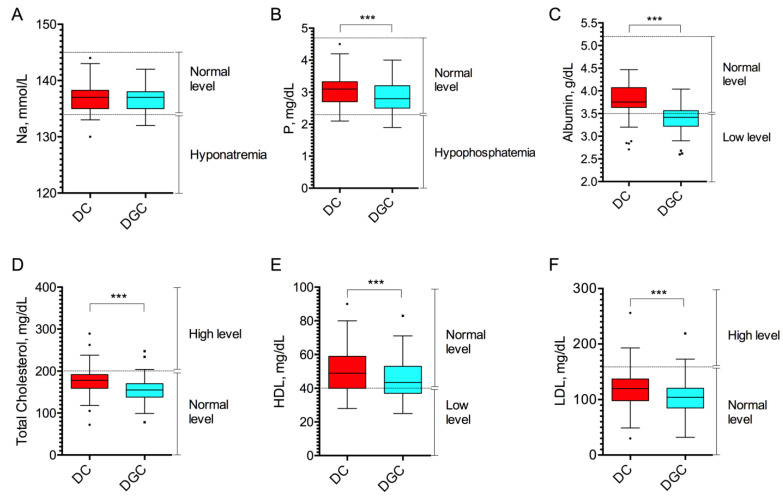
Biochemical analysis of plasma samples. Plasma levels of sodium (Na, (**A**)), phosphorus (P, (**B**)), albumin (**C**), total cholesterol (**D**), HDL (**E**), and LDL (**F**) fractions in plasma samples obtained by DC and DGC (n = 50). Data are shown as box and whisker plots. Dashed lines indicate the reference range for normal Na (134–145 mmol/L), P (2.3–4.7 mg/dL), albumin (3.5–5.2 g/dL) levels, and for cholesterol (>200 mg/dL), HDL (>40 mg/dL) and LDL (<160 mg/dL) associated with cardiovascular risk. *** *p* < 0.0001 by Wilcoxon test.

Instead, we analyzed the levels of sodium (Na^+^), a critical cation for maintaining fluid balance, blood pressure, plasma osmolarity, and phosphorus (P), involved in various body functions, including bone health, energy production, and cell membrane integrity. No difference in Na^+^ levels was observed between DC and DGC groups, with calculated mean concentrations of 137.1 ± 2.63 mmol/L (95% CI [136.4–137.9]) in the DC group and 136.9 ± 2.05 mmol/L (95% CI [136.3–137.5]) in the DGC group (*p* = 0.31, Figure 3A). P was slightly reduced in the DGC group, which presented a mean concentration of 2.84 ± 0.50 mg/dL (95% CI [2.69–2.98]), as compared to the DC group exhibiting a mean concentration of 3.09 ± 0.51 mg/dL (95% CI [2.94–3.23]; *p* < 0.0001, Figure 3B).

Finally, albumin and cholesterol levels were dosed to assess the impact of DC vs. DGC separation techniques on protein components and key biomarkers of metabolic health in plasma samples. The results showed that DGC samples had significantly lower levels of albumin, with an average concentration of 3.37 ± 0.29 g/dL (95% CI [3.29–3.46]), compared to 3.76 ± 0.39 g/dL (95% CI [3.65–3.87]) in samples obtained by DC (*p* < 0.0001, Figure 3C). Similarly, total cholesterol levels were lower in the DGC-treated plasma, with an average concentration of 156.1 ± 30.75 mg/dL (95% CI [147.4–164.9]), vs. 177.6 ± 37.24 mg/dL (95% CI [167.0–188.1]) in the DC group (*p* < 0.0001, Figure 3D). Subsequently, we analyzed the HDL and LDL fractions separately. For HDL, often referred to as “good cholesterol” due to its role in removing excess cholesterol from the bloodstream and transporting it to the liver for excretion, the DC technique showed an average concentration of 51.58 ± 13.34 mg/dL (95% CI [47.59–55.17]), while the DGC method yielded a lower concentration of 45.92 ± 11.99 mg/dL (95% CI [42.51–49.33]; *p* < 0.0001, Figure 3E). Regarding LDL, commonly known as “bad cholesterol” due to its association with an increased risk of atherosclerosis and cardiovascular diseases, the average values were 119.2 ± 37.40 mg/dL for DC and 104.1 ± 31.73 mg/dL (95% CI [95.06–113.1]) for DGC method (*p* < 0.0001, Figure 3F).

The comparison of qualitative parameter distributions between the DC and DGC groups revealed both similarities and notable divergences (Table 2). For Na^+^ and P, the majority of values fell within the reference range of normality, with no statistically significant differences observed between groups (*p* = 1.00 for Na^+^ and *p* = 0.06 for P). In contrast, albumin levels showed a marked disparity: 84% of samples in the DC group were within the normal range, compared to only 32% in the DGC group (*p* < 0.0001). An opposite trend was observed for total cholesterol, where a higher proportion of DGC group samples (94%) fell within range, compared to 82% in the DC group—a difference that was also statistically significant (*p* = 0.02). These results underscore the impact of DGC isolation method on specific biochemical parameters, i.e., albumin and total cholesterol, suggesting method-dependent variability in plasma composition.

### 2.3. Correlation Analysis of the Results

Correlation analysis was first conducted to explore the relationship between pre-analytical variables (hemolysis, lipemia, and icterus) and qualitative biochemical parameters analyzed in both the DC and DGC-treated samples (see Supplementary Table S1). HS showed a significant negative correlation with Na^+^ levels in both groups (DC: r = −0.482, *p* = 0.0004; DGC: r = −0.421, *p* = 0.002), suggesting that hemolysis may interfere with accurate Na quantification. A weak but significant positive correlation between HS and albumin was also observed in the DGC group (r = 0.316, *p* = 0.025), as well as with platelet counts (r = 0.349, *p* = 0.013), whereas no significant associations were detected in the DC group. Lipemia showed positive correlation with total cholesterol and LDL in both DC (r = 0.441 and 0.476; *p* = 0.001 and 0.0005, respectively) and DGC samples (r = 0.509 and 0.502; *p* = 0.0002 for both). Conversely, HDL levels were negatively correlated with lipemia in both groups (DC: r = −0.334, *p* = 0.018; DGC: r = −0.351, *p* = 0.014). No statistically significant correlations were found between bilirubin and any of the tested parameters in either group.

Then, to assess the consistency between the two separation methods, a correlation analysis was performed comparing the quality parameters measured in the samples obtained through DC and DGC methods. The analysis revealed strong positive correlations for most of the biochemical parameters tested, including triglycerides, bilirubin, Na^+^, P, total cholesterol and cholesterol fractions (HDL, LDL). Among all analytes considered, albumin showed the lowest correlation coefficient (r = 0.776), further suggesting a potential issue in the reliability of albumin measurement when using the DGC method. The results of the correlation analysis are reported in Table 3.

To further evaluate the agreement between DC and DGC methods, Bland–Altman analysis was performed for each biochemical parameter. The results demonstrated a generally good concordance between the two techniques, with the differences between paired measurements falling within the limits of agreement in more than 94% of the samples for all the analytes. However, a systematic bias was observed, as the differences (DC–DGC) were consistently greater than zero, indicating that the DC-derived values were systematically higher than those obtained with the DGC method. This trend was evident across nearly all tested parameters; Na was the only exception, showing a mean difference close to zero, thus suggesting negligible bias for this specific analyte. The Bland–Altman plots illustrating these findings are provided in Supplementary Figure S3.

## 3. Discussion

In the field of translational and biomarker research, the lack of standardization in plasma separation protocols represents a critical challenge to the reproducibility and comparability of results across studies [11,20]. Many published papers fail to clearly report how the starting plasma was obtained, leading to a “black box” in the preanalytical phase. For instance, in recent reviews on blood-based biomarkers, substantial heterogeneity is noted in the reported sample handling, and the omission of detailed descriptions of plasma isolation is frequently flagged as a source of bias in downstream measurements [5,21,22]. However, pre-analytical sample handling is increasingly recognized as a major source of variability and bias in biomarker studies.

Recognizing these gaps, our study aimed to compare two different plasma separation methods, by assessing their impact on the quality of isolated plasma for the accuracy of downstream measurements. While direct centrifugation (DC) is a straightforward method for rapid plasma isolation, density gradient centrifugation (DGC) may be in principle preferred when co-isolating PBMCs from the blood sample is required, as it optimizes cell separation in a single solution [23,24,25,26]. By comparing DC and DGC methods, we aimed to assess whether DGC may provide plasma of comparable quality to that obtained by DC, allowing the simultaneous biobanking of both plasma and PBMCs following a unique workflow for both blood derivatives.

Our findings indicate that preanalytical nonconformities, such as hemolysis, icterus, and lipemia, occurred with similar frequency in samples processed using the DC and DGC methods, suggesting no substantial difference in the overall integrity of the sample due to the separation techniques. Both methods exhibited some level of red blood cells (RBC) rupture, but the DGC method tended to induce less hemolysis, which could improve sample integrity and enhance the reliability of downstream analyses. Levels of bilirubin, derived from hemoglobin metabolism, were also slightly reduced in DGC samples as compared to DC. A plausible explanation lies in the lower centrifugation speed employed during the first centrifugation step in the DGC method, that may exert reduced mechanical stress on RBC membranes, thus decreasing the likelihood of cell lysis and subsequent release of hemoglobin into plasma [27,28].

Our results also showed that the DGC method led to higher platelet contamination. According to MISEV guidelines, platelet contamination in plasma samples poses a significant challenge for accurate analysis focused on circulating extracellular vesicles (EVs) [7]. Platelets are known to release microvesicles upon activation, which can contaminate the samples and confound results when analyzing EVs and EV-related assays, as they can be mistakenly identified as other types of EVs, leading to inaccurate quantification and characterization [29,30].

Observing the results regarding electrolytes, we found that P levels were slightly lower in DGC group. A similar reduction with DGC was already observed in existing literature, which aligns with our findings [22]. However, a limitation of our study was that we didn’t evaluate the impact of the isolation technique on calcium and potassium levels, due to the use of K3EDTA tubes for blood collection. EDTA acts as a potent chelator of divalent cations, including calcium, and can also influence potassium levels [31]. Consequently, any measurements of these electrolytes in EDTA-anticoagulated samples would not accurately reflect their physiological concentrations in vivo, potentially leading to misleading interpretations. Our focus remained on other plasma parameters that are reliably measured despite EDTA’s presence, ensuring the integrity and validity of our comparative analysis of the two plasma isolation methods.

A marked difference between the two plasma separation methods was observed in the concentration of key protein components, including albumin and cholesterol. In particular, employing DGC significantly reduced the levels of albumin and cholesterol, including both HDL and LDL fractions. These are critical biomarkers in many clinical assessments, and variations in their concentration can impact the interpretation of metabolic and disease-related tests [32]. An accurate lipid profile is essential for assessing cardiovascular health and lipid metabolism especially in patients under treatment [33].

Existing literature has extensively documented alterations in plasma components such as cholesterol and albumin in cancer patients compared to healthy individuals. For instance, low preoperative serum albumin has been associated with systemic inflammation and poor nutritional status, which are known negative prognostic indicators in breast cancer patients [34,35]. Similarly, alterations in HDL and LDL levels have been linked to tumor aggressiveness, response to therapy, and risk of recurrence [36,37].

Previous studies have analyzed how different centrifugation conditions influence plasma proteomics. It has been observed that variables such as centrifugation speed, duration, and the use of a brake can alter the plasma protein composition, affecting the outcomes of proteomic analyses [38]. Our data are consistent with these studies, emphasizing how methodological differences in plasma preparation may significantly influence albumin and lipid measurements. Consequently, the choice of separation method should be carefully considered as it may affect the reliability and reproducibility in proteomic studies.

Despite some notable differences highlighting the impact of the separation method on general plasma quality, we observed a high level of correlation and agreement between DC and DGC for most of the biochemical parameters tested. This result proves that, in principle, both approaches maintain inter-patient variability. Nevertheless, it is essential to notice that the absolute levels detected in matched samples are different when the two approaches are used, with generally higher levels when DC is used. The interpretation of research analyses thus requires precise knowledge of the separation method used for the plasma separation as the lack of specification could result in a significant misinterpretation of the data. It is notable to observe that for albumin and cholesterol the percentage of samples out of the optimal reference range was found significantly different when using DGC or DC separation method, and that albumin measurements showed the lowest correlation coefficient when comparing the two methods. This finding further indicates that caution may be warranted when using DGC, as this technique significantly alters the overall plasma composition.

We acknowledge as a limitation of this study the absence of a healthy control group, which would be valuable in future research to assess the impact of the separation method on the accuracy of diagnostic and prognostic analyses made on plasma. Including healthy volunteers in future studies would provide a baseline for understanding how disease states influence the separation process and the resulting measurements, which was not the focus of the present study.

Furthermore, while the current study primarily focused on the general quality assessment of plasma samples within the biobanking context, we acknowledge that analytes such as circulating tumor DNA (ctDNA) and extracellular vesicles (EVs) play a crucial role in the future applications of liquid biopsy. Given that these analytes are present at much lower concentrations than the those measured here, their accurate quantification requires more sensitive and selective approaches [39,40]. Future research studies are warranted to explore this aspect further, with specialized analyses to determine the extent to which different separation techniques impact the measurement and clinical relevance of specific tumor biomarkers.

## 4. Materials and Methods

### 4.1. Patient Population

A total of 50 consecutive breast cancer patients referred to the EUSOMA-accredited Breast Unit of Istituti Clinici Scientifici Maugeri (Pavia, Italy) who have signed a written informed consent for biobanking from September to December 2024 were included in the study. All the patients were female, had fasted overnight and were candidate to breast surgery. The main baseline characteristics of the study cohort are summarized in Table 4.

### 4.2. Blood Collection and Plasma Isolation

Blood samples (approximately 6–10 mL of peripheral venous blood per patient) were collected by qualified nursing staff in EDTA-treated Vacutainer K3 tubes prior to surgical intervention. Blood tubes were gently inverted, sent at room temperature (RT) to the Institutional Biobank “Bruno Boerci” (Istituti Clinici Scientifici Maugeri IRCCS, Pavia, Italy) and processed within 2 h. All human samples were pseudonymized and stored according to standard operating procedures (SOPs) adopted by the Biobank. Individual samples were divided into two aliquots (3–5 mL per aliquot) that were processed according to two different plasma isolation protocols for a comparative purpose: density gradient centrifugation (DGC) and direct centrifugation (DC). For the former, Histopaque^®^-1077 density gradient (Sigma-Aldrich, St. Louis, MO, USA, –10771) was brought into RT before use. An aliquot of blood was slowly layered on top of an equal volume of Histopaque into a 15 mL polypropylene tube and put into a swinging bucket centrifuge for density centrifugation at 400× *g* for 30 min at RT, without acceleration and brake, to achieve a clear separation of plasma, peripheral blood mononuclear cells (PBMCs) and erythrocytes. For the DC procedure, one aliquot of blood was centrifuged at 2000× *g* at RT for 10 min. The obtained upper layer of plasma from both DC and DGC methods was separately transferred into a 2 mL tube and centrifuged at 2500× *g* for 10 min at 4 °C to remove platelets, as previously described [15,41,42,43,44]. Plasma was aliquoted (0.5 mL/aliquot), transferred in cryogenic vials, labeled with appropriate de-identified labels and freshly used for quality control. Following the Biobank’s SOPs, any leftover sample after analysis was frozen at −80 °C.

### 4.3. Spectrophotometric Analysis

To assess the quality of plasma samples obtained using DGC or DC isolation methods, we performed visual and spectrophotometric evaluation of hemolysis and lipemia. Ultraviolet-visible (Uv-Vis) absorbance measurements were carried out using a NanoDrop 2000c Spectrophotometer (ThermoFisherScientific, Waltham, MA, USA) by applying 2 µL of sample on the micro-volume pedestal. The spectra were acquired and absorbances at λ = 414 nm and λ = 385 nm were recorded as parameters reflecting free hemoglobin and lipemic samples, respectively. A lipemia-independent hemolysis score (HS) was calculated using the following formula: $HS=\left(Absorbance\;414\;nm-Absorbance\;385\;nm\right)+0.16*Absorbance\;385\;nm$ as previously described [16]. Samples with an HS > 0.25 were classified as hemolytic as previously described [17].

### 4.4. Hemocytometric Analysis

Samples were analyzed for cellular debris and platelet contamination by automated haematology analyzer Sysmex XN (Sysmex, Kobe, Japan) in the clinical laboratory of Istituti Clinici Scientifici Maugeri (Pavia, Italy). The analyser Sysmex XN uses sheath flow impedance technology to measure platelet and red blood (RBC) cells (the RBC channel), as well as fluorescence flow cytometry for the measurement and differentiation (mononuclear polymorphonuclear leukocytes) of white blood (WBC) cells (the WBC channel). XN-L Check, with three different levels, were used as quality controls.

### 4.5. Biochemical Analysis

Biochemical analyses, conducted by clinical laboratory, were performed on plasma specimens. The clinical chemistry and electrolyte parameters assessed on the Alinity c system (Abbott Diagnostics, Abbott Park, IL, USA) included: albumin, total bilirubin, triglycerides, total cholesterol, low-density lipoproteins (LDL) and high-density lipoproteins (HDL), sodium and phosphorus (Na^+^; P).

### 4.6. Statistical Analysis

Statistical analyses were performed using OriginPro software (Version 2024b) (OriginLab, Northampton, MA, USA) and GraphPad Prism software (Version 6.0e). Normality was first assessed by Shapiro–Wilk test, followed by either a parametric (two-sample *t*-test) or non-parametric test (Wilcoxon matched-pairs signed rank test), as appropriate, to compare DGC and DC groups. Chi-square (and Fisher’s exact) tests were used to assess the relationship between nonconformity (e.g., hemolytic, icteric and lipemic samples) and the plasma separation method. Correlation coefficient (Pearson in case of normal data, Spearman for non-normal data) was calculated to assess the association between analytical measures obtained with DC or DGC method. Variables were presented as means, standard deviations and confidence intervals of 95%, or as absolute numbers and percentages. Statistical significance was defined as *p* < 0.05 (two-tailed). The Bland–Altman method was used to assess the agreement between the measurements obtained through the two methods

## 5. Conclusions

This study demonstrates that plasma isolation technique significantly influences the molecular and biochemical sample quality. DC provides plasma with lower platelet contamination and more consistent biochemical profiles as compared to DGC, supporting higher molecular integrity. Aligning biobank workflows with specific research goals and adhering to international recommendations—including reporting separation methods and assessing preanalytical variables—ensure that samples are not only preserved but fully “research-ready”, enabling reproducible biomarker discovery and reliable translational oncology studies.

## Figures and Tables

**Table 1:** Comparison of non-conformity values (hemolysis, lipemia, and icterus) between DC and DGC methods (n = 50). No significant difference was found between groups (Fisher’s exact test).

Type of Non-Conformity	DC (n, %)	DGC (n, %)	*p*-Value
Hemolysis	4, 8%	5, 10%	0.81
Lipemia	3, 6%	1, 2%	0.28
Icterus	0, 0%	0, 0%	1.00

**Table 2:** Comparison of in-range and out-of-range distributions of qualitative parameters measured as number and percentage in DC and DGC groups. The reference ranges used in this study were derived from the clinical laboratory reports and correspond to those routinely adopted in clinical practice for risk profiling and patient assessment.

Parameter (Reference Range of Normality)	DC (n = 50)		DGC (n = 50)		*p*-Value DC vs. DGC
	In Range	Out-of-Range	In Range	Out-of-Range	
Na (134–145 mmol/L)	47 (94%)	3 (6%)	48 (96%)	2 (4%)	1.00
P (2.3–4.7 mg/dL)	49 (98%)	1 (2%)	43 (86%)	7 (14%)	0.06
Albumin (3.5–5.2 g/dL)	42 (84%)	8 (16%)	16 (32%)	34 (68%)	<0.0001
Total Chol. (<200 mg/dL)	41 (82%)	9 (18%)	47 (94%)	3 (6%)	0.02
HDL (>40 mg/dL)	40 (80%)	10 (20%)	32 (64%)	18 (36%)	0.08
LDL (<160 mg/dL)	44 (88%)	6 (12%)	48 (96%)	2 (4%)	0.14

**Table 3:** Correlation analysis of biochemical parameters measured in matched samples obtained with DC and DGC method.

Parameter	Correlation Coefficient [95% CI]	*p* Value
Na^+^	0.824 [0.708–0.897]	<0.0001
P	0.966 [0.911–0.971]	<0.0001
Albumin	0.776 [0.629–0.869]	<0.0001
Total Cholesterol	0.949 [0.911–0.971]	<0.0001
HDL	0.956 [0.922–0.975]	<0.0001
LDL	0.945 [0.903–0.969]	<0.0001
Triglycerides	0.965 [0.937–0.980]	<0.0001
Bilirubin	0.978 [0.961–0.988]	<0.0001

**Table 4:** Baseline characteristics of the patient population.

Parameter	Value (n = 50)
Age (mean ± SD)	63.26 ± 15.76
BMI (mean ± SD)	24.71 ± 3.93
Tumor grade (n, %)	
G1	7, 14%
G2	28, 56%
G3	14, 28%
Not specified	1, 2%

## Data Availability

Raw data are available at https://doi.org/10.5281/zenodo.17314452.

## Supplementary Materials

The following supporting information can be downloaded at: https://www.mdpi.com/article/10.3390/ijms262110281/s1.

## Abbreviations

SOPs, standard operating procedures; ISEV, International Society for Extracellular Vesicles; ISLB, International Society for Liquid Biopsy; DGC, density gradient centrifugation; DC, direct centrifugation; PBMCs, peripheral blood mononuclear cells; Uv-Vis, Ultraviolet-visible; HS, haemolysis score; Na^+^, sodium; P, phosphorus; HDL, high density lipoproteins; LDL, low density lipoproteins; MISEV, Minimal information for studies of extracellular vesicles; RT, room temperature.
