# Supplementary material for: Influence of Isolation Techniques on the Quality of Plasma Samples: Implications for Cancer Biobanking
Source: Int J Mol Sci. 2025 Oct 22;26(21):10281. doi: 10.3390/ijms262110281 (PMC12610171; doi:10.3390/ijms262110281)
Supplement: Supplementary file 1 [file ijms-26-10281-s001.zip › ijms-3905879-supplementary.pdf]

Supplementary Materials

Figure S1. Comparison of plasma yield between DC and DGC protocols, expressed as a percentage of total blood volume. Statistical significance was assessed using Wilcoxon matched-pairs signed rank test.

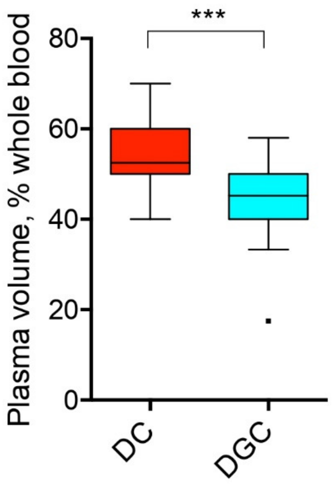

Figure S2. Frequency distribution of platelet counts in plasma samples isolated using the DC and DGC methods. The histogram illustrates the relative frequency of platelet values across defined intervals (<10, 10-20, >20 for each group (n=50).

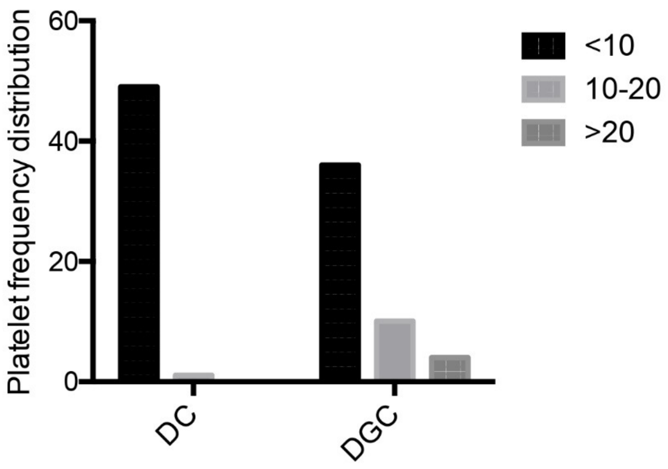

Figure S3. Bland-Altman plot between DC and DGC for preanalytical parameters. The black horizontal line represents the mean differences between paired measurements (DC-DGC). The red dashed lines indicate the upper and lower limits of agreement, calculated as the mean difference ± 1.96 times the standard deviation of the differences. These limits define the range within which approximately 95% of the differences between the two measurements are expected to fall.

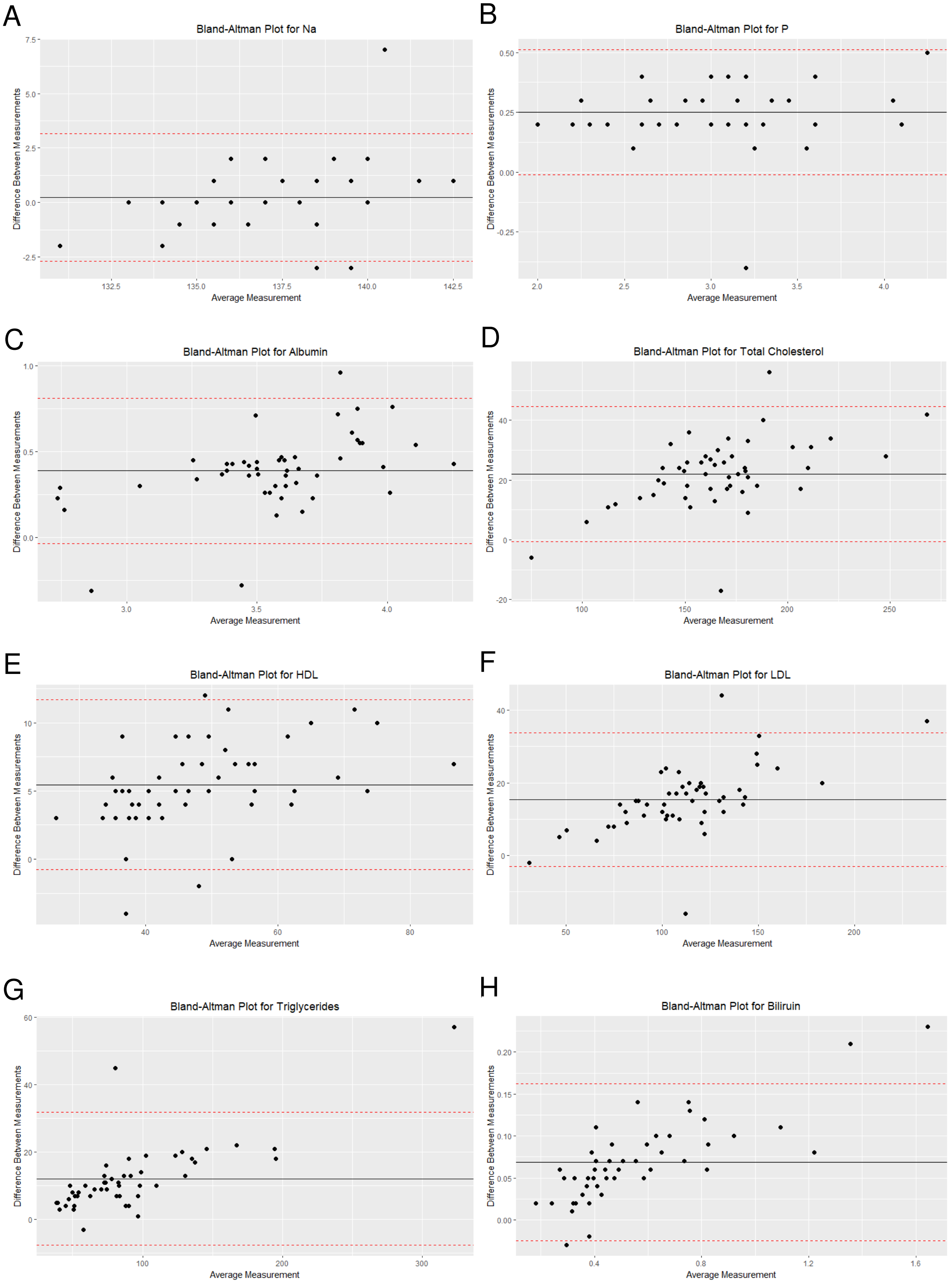

A
Bland-Altman Plot for Na
B
Bland-Altman Plot for P
C
Bland-Altman Plot for Albumin
D
Bland-Altman Plot for Total Cholesterol
E
Bland-Altman Plot for HDL
F
Bland-Altman Plot for LDL
G
Bland-Altman Plot for Triglycerides
H
Bland-Altman Plot for Biliruin
Difference Between Measurements
Average Measurement

Table S1. Correlation analysis of preanalytical variables (hemolysis, lipemia, icterus) and qualitative parameters across the two sample sets.

	Parameter	DC		DGC	
		Corr. Coeff	p-value	Corr. Coeff	p-value
HS	Na	- 0.482	0.0004***	- 0.421	0.002**
	P	0.011	0.939	0.026	0.856
	Albumin	0.243	0.089	0.316	0.025*
	Tot. Cholesterol	0.086	0.550	0.203	0.157
	HDL	0.194	0.177	0.233	0.103
	LDL	0.102	0.480	0.140	0.331
	Platelets	0.039	0.785	0.349	0.013*
	Bilirubin	- 0.138	0.339	0.075	0.604
	Triglycerides	- 0.107	0.459	- 0.003	0.983
Lipemia	Na	- 0.114	0.432	0.067	0.647
	P	0.247	0.084	0.224	0.122
	Albumin	0.051	0.725	0.067	0.646
	Tot. Cholesterol	0.441	0.001**	0.509	0.0002***
	HDL	- 0.334	0.018*	- 0.351	0.014*
	LDL	0.476	0.0005***	0.502	0.0002***
	Platelets	- 0.045	0.757	0.045	0.758
	Bilirubin	0.211	0.142	0.239	0.097
	HS	- 0.107	0.459	- 0.003	0.983

Icterus	Na	- 0.044	0.758	0.039	0.789
	P	- 0.213	0.138	- 0.138	0.335
	Albumin	0.085	0.557	0.098	0.491
	Tot. Cholesterol	0.054	0.709	0.106	0.465
	HDL	- 0.006	0.968	- 0.038	0.794
	LDL	0.104	0.471	0.106	0.465
	Platelets	- 0,240	0.093	0.239	0.097
	Triglycerides	0.211	0.142	- 0.024	0.869
	HS	- 0.138	0.339	0.075	0.604
